# An examination of objectively-measured sedentary behavior and mental well-being in adults across week days and weekends

**DOI:** 10.1371/journal.pone.0185143

**Published:** 2017-09-21

**Authors:** Ann-Marie Gibson, David J. Muggeridge, Adrienne R. Hughes, Louise Kelly, Alison Kirk

**Affiliations:** 1 Physical Activity for Health, School of Psychological Sciences and Health, University of Strathclyde, Glasgow, United Kingdom; 2 Department of Exercise Science, California Lutheran University, Thousand Oaks, CA, United States of America; Texas Technical University Health Sciences Center, UNITED STATES

## Abstract

**Background:**

Limited research has explored the links between sedentary behaviour, mental health and quality of life. This study examines objectively measured sedentary behaviour and perceived mental health and quality of life across week days and weekends.

**Methods:**

42 adults (19M, 23F; mean age 38yrs (range 18–67) & BMI 24.8kg/m^2^ (range 18.7–33.8) wore an activPAL monitor 24h/day for one week and completed the Hospital Anxiety and Depression Scale (HADS) and SF12 Health Survey. Average weekday and weekend day sitting time was computed. Differences between sitting (Group 1 = <8hrs/day, Group 2 = 8–10 hrs/day, Group 3 = >10hrs/day) and components of the HADS and SF12 health survey were examined using an ANCOVA with a measure of physical activity (step count) included as a covariate.

**Results:**

Average sitting time on a weekday was 9hrs 29mins (range 5hrs 52mins to 12hrs 55mins) and 8hrs 59mins (range 4hrs, 07mins to 14hrs, 40mins) on a weekend day. There was a main effect (p<0.05) for weekday sitting time on total anxiety and depression (HADS) and mental health and vitality (SF12). Planned contrasts identified individuals in group 1 had lower anxiety and depression and higher mental health and vitality scores than individuals in groups 2 or 3 (p<0.05). No difference was found between individuals in group 2 and group 3 (p>0.05). No main effects were found for weekend sitting (p>0.05).

**Conclusions:**

Weekday sitting time below 8 hours/day is associated with better perceived mental health and quality of life.

## Introduction

Mental illness is an important public health priority [1,2]. There is a wealth of research evidence suggesting that regular physical activity is beneficial for individuals with mental health disorders [3,4] yet an additional lifestyle factor that may be important is sedentary behavior. Sedentary behavior can be defined as any waking activity characterised by an energy expenditure ≤ 1.5 metabolic equivalents and a sitting or reclining posture [5]. It is now well established that sedentary behavior is independent of an individual’s physical activity levels in their associations with health [6,7]. Epidemiological studies have consistently shown that spending excessive time engaged in sedentary behaviors may have a negative impact on several physical health outcomes yet the research examining the relationship between sedentary behavior and mental health outcomes (e.g., depression, anxiety, quality of life) is somewhat sparse with limitations in the assessment and classification of sedentary behavior.

It has been suggested that adults spend between 6–10 hours sitting each day [8,9]. Recent data has highlighted that for the majority of working adults, time spent sitting during the working day is more likely to contribute to overall time spent sitting than the time spent sitting during leisure time [10]. Examining sitting time during the working week compared to at weekends could provide additional insight into the links with mental health and well-being, particularly in employees. It could be suggested that there may be different effects of weekday and weekend day sitting due to the lack of volition individuals have over their sitting behaviours within the workplace, in comparison to their leisure time. Current proposed mechanisms as to why engaging in prolonged periods of sedentary behavior could lead to poorer mental health include: i) the social withdrawal hypothesis [11] ii) the time displacement hypothesis [12] and iii) the involvement of inflammatory markers [13]. The majority of cross-sectional evidence suggests that watching TV is associated with poorer mental health [14,15] yet the evidence for other sedentary behaviors, for example computer use, is mixed and it has been argued that the association with mental health is often dependent on the purpose and content of the computer use [16]. Longitudinal evidence has shown that engaging in more TV watching and computer use at baseline predicts a greater risk of depression at follow-up [17] and that depressive symptoms at baseline are predictive of more TV viewing at follow-up [18]. A recent meta-analysis examining sedentary behavior and the risk of depression across 13 cross-sectional and 11 longitudinal studies [19] suggested that different types of sedentary activities may have differing associations with mental health.

It could also be suggested that sedentary behavior influences different types of mental illness in varying degrees, yet the majority of studies focus on depression. In a recent systematic review, Teychenne, Costigan and Parker [20] suggested that a positive relationship may exist between overall sitting time and anxiety yet there was inconsistent evidence for other types of sedentary behavior (e.g., TV viewing, computer use) and the links with the risk of anxiety. As highlighted in this review and previous research, an important identifiable limitation in the research to date is the reliance on self-report items to assess sedentary behavior, often only measuring one type of sedentary behavior, usually TV viewing, which is not considered an accurate measure of total sedentary time. The aim of the study was to examine objectively measured sedentary behavior and mental health and quality of life across both week and weekend days in adults.

## Methods

All participants gave written informed consent and ethical approval was obtained for the study from the School of Psychological Sciences and Health’s Ethics Committee at the University of Strathclyde.

### Participants

Forty-two adults (19M aged 21–67 years; 23F aged 18–52 years; mean age = 38.0 ± 11.5 years, mean BMI = 24.8 ± 2.0 kg/m^2^) who worked a minimum of 30 hours per week were recruited for the study through e-mail, posters and word of mouth.

### Procedures

Participants provided informed consent, completed a demographic questionnaire and stature and mass were also assessed. Participants were then asked to wear an activPAL on their left thigh for 24 hours/day for seven consecutive days. Participants were given a wear diary, where they were asked to record what time they woke up and when they went to sleep and to record if the activPAL was removed and the reasons for removal. After one week, participants returned the activPAL and the wear diary to the researchers, and completed the Hospital Anxiety and Depression Scale (HADS) and the Short Form-12 questionnaire (SF-12). Sedentary time was calculated as monitor recorded sitting or lying time minus wear diary recorded sleep time. Wear diary sleep time was verified against monitor recorded activity.

### Measures

#### Depression and anxiety

The HADS [21] is a 14-item self-report questionnaire used to assess anxiety and depression, which requires participants to recall how they have felt in the previous week. Scores are summed where a score of 0–7 signifies no presence of clinical symptoms; 8–10 indicates mild symptoms; 11–14 indicates moderate symptoms and a score of 15–21 indicates severe symptoms.

#### Health-related quality of life

The SF-12 [22] is 12-item self-report questionnaire used to assess aspects of overall health-related quality of life. There are eight subscales relating to physical and mental health (general health; physical functioning; emotional role functioning; physical role functioning; bodily pain; mental health; vitality and social functioning). A higher % score on the SF-12 subscales represents a higher level of physical and mental health.

#### Physical measurements

Stature was recorded using a Stadiometer (Model 225, Seca Ltd) and body mass was assessed using precision scales (Model 770, Seca Ltd).

#### Sedentary behavior

Sedentary behavior was measured using an activPAL mini, an inclinometer-based activity monitor which can directly identify periods of sitting/lying, standing and stepping. The activPAL has been shown to be 32.4% more accurate than actigraphs with 99.1% accuracy for sitting, standing, and slow walking [23]. A minimum of three days of data were required to be included in analysis (including at least one weekend day), which is in accordance with similar studies [24, 25].

### Data analysis

Average total daily time spent in sedentary, standing and stepping activity, in addition to total daily step counts and sit to stand transitions, were computed from the activPAL software for 1) weekdays and 2) weekend days. Paired t-tests were used to examine differences in total daily time spent in sedentary, standing and stepping activity, in addition to total daily step counts and sit to stand transitions between weekdays and weekend days. Participants were grouped according to time spent sitting based on the overall group mean. In relation to weekday sedentary behavior (n = 42), 10 participants were in group 1; 14 participants were in group 2 and 18 participants were in group 3. In relation to weekend sedentary behavior (n = 39), 13 participants were in group 1; 12 participants were in group 2 and 14 participants were in group 3. Differences between sitting (Group 1 = <8hrs/day, Group 2 = 8–10 hrs/day, Group 3 = >10hrs/day) and components of the HADS and SF12 were examined using an ANCOVA with a measure of physical activity (step count) included as a covariate. Partial eta-squared (η_p_^2^) effect sizes were used to evaluate the strength of association for between group differences. Values of 0.01–0.03, 0.06–0.09 and >0.14 indicate a small, medium and large effect, respectively [26].

## Results

### Weekday and weekend day sedentary behavior

The mean hours spent in sedentary, standing and stepping activity, in addition to total daily step counts and sit to stand transitions during weekdays and weekend days, are reported in Table 1. Paired t-tests revealed that average sedentary time on weekdays was higher (p ≤ 0.05) than weekend days. Time spent standing was higher (p ≤ 0.05) at weekends than weekdays. Time spent stepping was similar between weekdays and weekend, however step count was lower (p ≤ 0.05) on weekend days. The number of sit to stand transitions was higher (p ≤ 0.05) during weekdays than on weekend days.

**Table 1:** Sedentary behavior during weekdays and weekend days.

	Weekday (n = 42)		Weekend day (n = 39)	
	Mean	SD	Mean	SD
**Sitting (hrs)**	9.5*	1.7	9.0	2.4
**Standing (hrs)**	3.8*	1.4	4.0	2.0
**Stepping (hrs)**	1.7	0.6	1.6	0.7
**Step count**	8668*	3279	7897	3863
**Sit to stand transitions**	60*	23	54	21

* denotes significant difference between week and weekend days (p ≤ 0.05)

### Sedentary behavior and mental health

**Fig 1:**
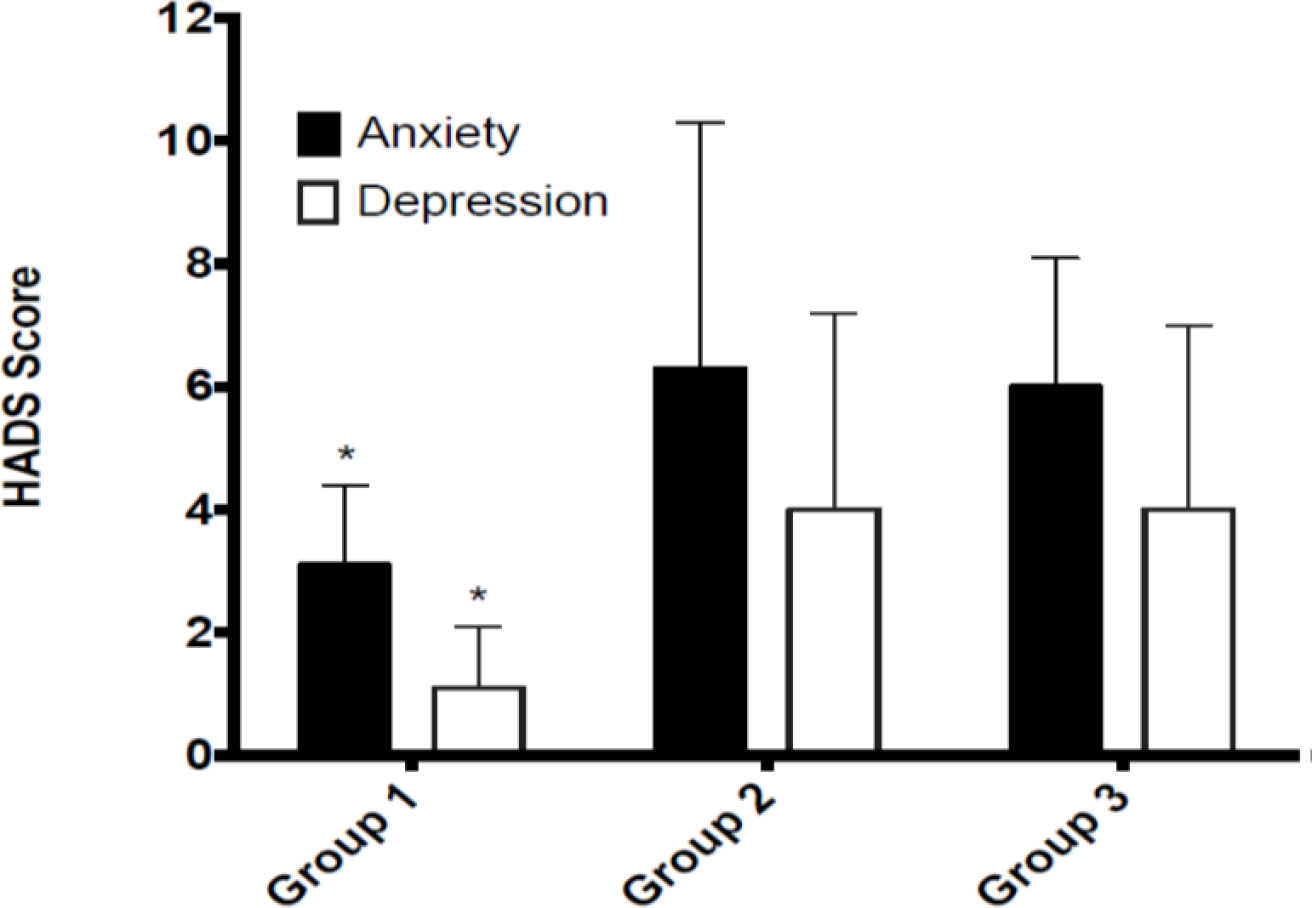
Weekday sedentary behavior group differences in levels of anxiety and depression.

**Fig 2:**
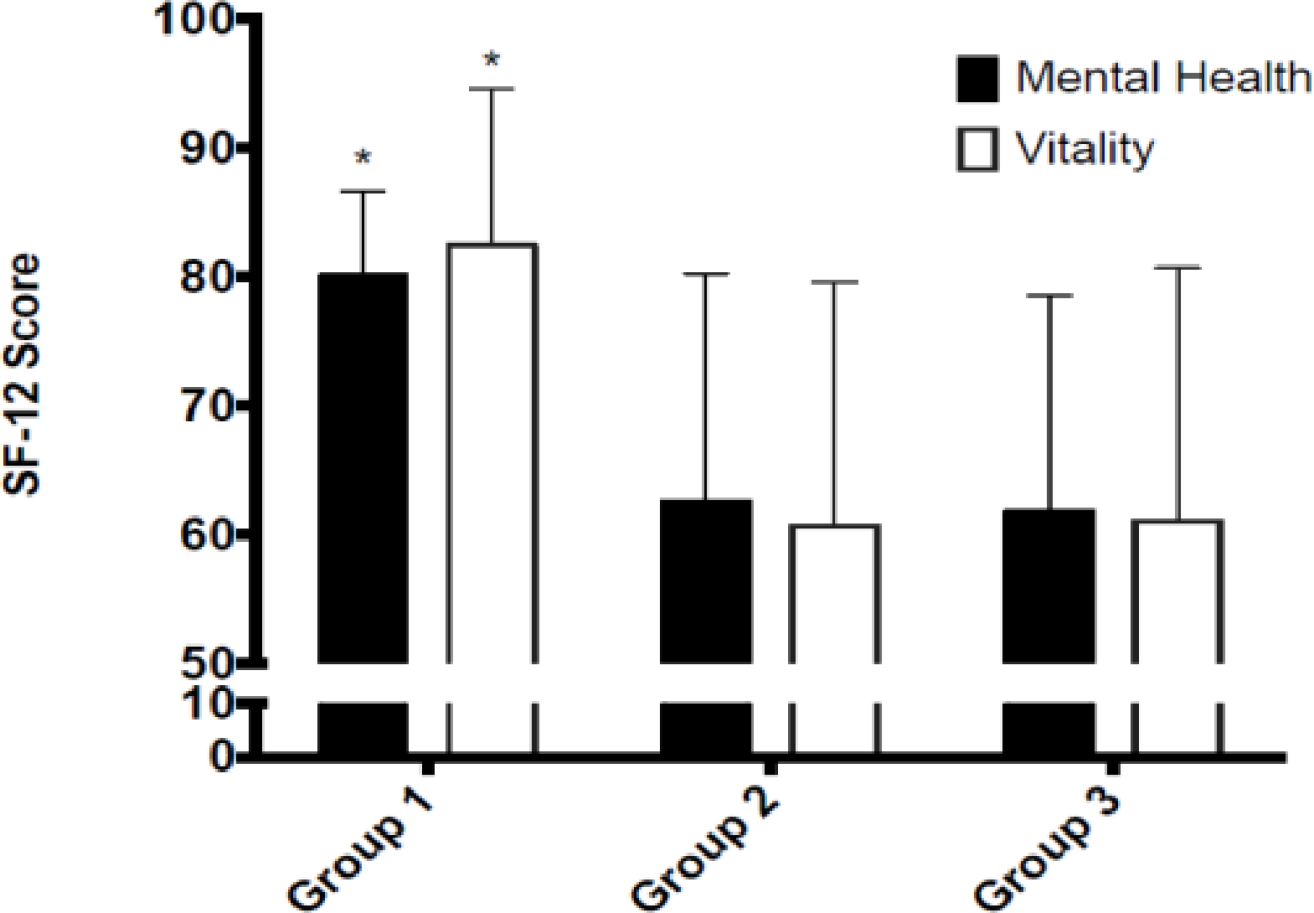
Weekday sedentary behavior group differences in mental health and vitality scores.

The mean (SD) values for anxiety and depression (HADS) and the eight subscales of the SF-12 are shown in Table 2. There was a main effect for weekday sitting time on anxiety (F_(1, 41)_ = 3.05, p = 0.040, ƞ^2^ = 0.18), depression (F_(1, 41)_ = 2.91, p = 0.047, ƞ^2^ = 0.16), mental health (F_(1, 41)_ = 3.50, p = 0.025, ƞ^2^ = 0.17) and vitality (F_(1, 41)_ = 3.70, p = 0.020, ƞ^2^ = 0.22). Planned contrasts identified individuals in group 1 had lower anxiety and depression (Fig 1) and higher mental health and vitality scores (Fig 2) than individuals in groups 2 or 3 (p ≤ 0.05). No differences were found between individuals in group 2 and group 3 (p ≤ 0.05). No main effects were found for weekend sitting (p ≥ 0.05) across any of the mental health outcome variables.

**Table 2:** Mental health variables according to sedentary behavior groups.

	Weekday (n = 42)			Weekend day (n = 39)		
	Mean (SD)			Mean (SD)		
	Group 1	Group 2	Group 3	Group 1	Group 2	Group 3
Anxiety	3.1	6.3	6.0	6.0	3.7	4.6
	(1.3)	(4.0)	(2.1)	(2.7)	(2.6)	(2.4)
Depression	1.1	4.0	4.0	3.2	2.2	2.1
	(1.0)	(3.2)	(3.0)	(2.8)	(1.4)	(1.8)
General health	67.6	75.0	67.0	72.5	60.0	75.0
	(15.1)	(19.3)	(21.4)	(11.7)	(16.5)	(12.0)
Physical functioning	94.1	95.0	86.6	93.8	88.3	93.8
	(6.2)	(4.1)	(8.9)	(4.0)	(7.6)	(2.8)
Role functional	90.0	92.9	88.9	100	81.8	100
(Physical)	(31.6)	(26.7)	(32.3)	(0.0)	(40.4)	(0.0)
Role functional	100	85.7	94.4	88.9	100	100
(Emotional)	(0.0)	(36.3)	(23.6)	(33.3)	(0.0)	(0.0)
Bodily pain	97.5	91.1	88.9	94.4	90.9	91.7
	(7.9)	(15.8)	(17.6)	(11.0)	(16.9)	(17.7)
Mental health	80.1	62.6	61.8	69.5	69.4	68.1
	(6.5)	(17.7)	(16.8)	(16.7)	(16.0)	(12.6)
Vitality	82.5	60.7	61.1	69.4	68.2	77.8
	(12.1)	(18.9)	(19.6)	(11.0)	(19.7)	(19.5)
Social functioning	95.0	91.1	84.7	97.2	88.6	88.9
	(15.8)	(21.0)	(29.9)	(8.3)	(20.5)	(33.3)

## Discussion

The aim was to examine objectively measured sedentary behavior and mental health and quality of life across both week and weekend days in adults. There were significant differences in sedentary behavior across weekdays compared to weekends. Participants spent more time sitting and less time standing during the week compared to the weekend yet this was accompanied by a significantly greater step count. The increase in time spent sitting during weekdays could be attributed to occupation-related behavior within the sample as all of the participants worked at least 30 hours during the week in variable occupations. Our findings contradict those reported in the Health Survey England [27] where adults self-reported spending 4.8 hours being sedentary on weekdays and 5.3 hours at weekends. Yet it should be noted that the higher values of sedentary behavior reported in the current study could be due to the use of an objective method to assess sedentary behavior more accurately. Our findings are similar to those reported in a study by Thorp et al. [10]. They assessed sedentary behavior objectively, comparing work days and non-work days, and found that on work days, participants spent 10.7 hours in sedentary activities yet 8.6 hours in sedentary activities on non-work days. These findings, along with those in the current study, provide support that the workplace could be a key setting for sedentary behavior and should be targeted for appropriate public health intervention strategies.

Understanding the association between varying levels of sedentary behavior on mental health outcomes was also examined. Findings indicated that those participants who engaged in less than 8 hours of sedentary behavior per day on weekdays had significantly lower levels of anxiety and depression compared with those who engaged in greater than 8 hours per day. Findings also indicated a large effect of sitting less than 8 hours per day on lower levels of anxiety and depression. These findings support conclusions drawn from systematic reviews [16,20] and a meta-analysis [19] examining the links between anxiety, depression and sedentary behavior. In support of the current findings, data from the meta-analysis indicated there was a significant association between sedentary behavior and risk of depression yet only three of the twenty studies included assessed sedentary behavior using an objective measure. Our findings add to the limited research examining the association between anxiety and sedentary behavior, contributing to only one known study [28] that used an objective measure of sedentary behavior.

In relation to aspects of health-related quality of life, findings indicated that those participants who engaged in less than 8 hours of sedentary behavior per day on weekdays had significantly higher levels of vitality and mental health compared with those who engaged in greater than 8 hours per day. Findings also indicated a large effect of sitting less than 8 hours per day on higher levels of vitality and mental health. There is limited research examining the links between aspects of health-related quality of life and sedentary behavior yet our findings support a prospective cohort study conducted with older adults [29]. They found significant increases in levels of vitality across decreased sitting time yet the measure of sedentary behavior was self-report and focused on sitting time during leisure activities as opposed to the waking day as in the current study. Understanding how and why sedentary behavior could influence mental well-being (e.g., depression, anxiety and vitality) is an area of research that is lacking and needs further attention.

A strength of the current study is the use of an objective measure to assess sedentary behavior across both weekdays and weekends and relate to a range of mental health outcomes in an adult population. Our findings highlighted that there were weekday and weekend differences in sitting time and that those participants who spent less than 8 hours per day sitting, were less depressed, anxious and had higher levels of vitality than those who spent over 8 hours sitting. Yet it is important to note that due to the cross-sectional nature of the study, it could be argued that the participants who were more depressed, anxious and had lower levels of vitality could be more prone to spending greater amounts of time sitting. Furthermore, this association was only evident during the weekdays. Contextual information regarding the type of sedentary activities participated in during measured sitting time and where sitting behavior occurred was not obtained. This is an identifiable limitation of the study, along with the cross-sectional nature of the data, making it difficult to infer cause and effect. This would have allowed us to further explore the workplace as a potential important context for sedentary behavior and mental health. Whilst our findings suggest a large positive effect for sitting less than 8 hours per day on improved mental well-being, we acknowledge a requirement for further research with larger groups of participants in order to translate our findings into meaningful recommendations. Our data indicates that reducing sedentary behavior during the working day could be important for improving employees’ mental health and quality of life. Therefore, encouraging workplace intervention studies to include mental health and quality of life outcome measures is warranted to explore the resultant effect of reducing sedentary behavior during the working day.

## Supporting information

S1 File Sedentary behavior and mental health excel data sheet. (XLSX)
